# How compatible are Western psychology and yoga psychology? Epistemology, concepts and localization

**DOI:** 10.3389/fpsyg.2025.1554014

**Published:** 2025-03-11

**Authors:** Stephan Schleim

**Affiliations:** ^1^Theory and History of Psychology, Faculty of Behavioral and Social Sciences, Heymans Institute for Psychological Research, University of Groningen, Groningen, Netherlands; ^2^Stephan Schleim Philosophy and Psychology, Amersfoort, Netherlands

**Keywords:** yoga, mindfulness, meditation, epistemology, ontology, pragmatism, consciousness

## Abstract

Mindfulness, meditation and yoga are very popular today. A large number of studies and meta-analyses have investigated the effectiveness of such practices for health, wellness and fitness. Yoga itself has repeatedly been described a science or science-based practice since the 20th century. This perspective addresses the question of the extent to which Western psychology and science are compatible with yoga psychology. To do this, we will first narrow down the meaning of “yoga,” namely on the basis of the classical *Yoga Sutras*, a text on yoga that is probably at least 1,600 years old. According to this system, yoga is a combination of ethical rules, postures, breathing exercises and meditative techniques. The emphasis here is on epistemology: Which sources are accepted for valid knowledge in this system? Ontology is then discussed in the broader context of Indian philosophy. In a further section, the conceptualization and localization of mental faculties is discussed. This perspective discusses assumptions from Indian schools of thought such as yoga, which seem difficult to reconcile with Western science. One way to resolve this conflict is to reinterpret the terms and statements of classical sources of Indian philosophy. While this would serve compatibility with science, it probably undermines the authenticity and inner core of the Indian philosophical systems addressed here.

## Introduction

Mindfulness, meditation and yoga exercises have become very popular. In 2024, for example, the United Nations proclaimed the 10th annual International Day of Yoga (June 21)^1^ and the 21st annual World Meditation Day (December 21).^2^ These initiatives focus on physical and mental health. In the words of the UN High Commissioner for Human Rights, Volker Türk: “Meditation builds resilience, clarity & empathy—tools for navigating life’s stressors.”^3^

The cultural and historical roots of such practices in India and other Asian countries are generally less well known, even though anthropologists, Indologists and religious scholars, for example, are conducting research in this area (e.g., Baier et al., 2018; Newcombe and O’Brien-Kop, 2021). In psychology and neuroscience, the cognitive and emotional abilities of long-term meditators from Buddhist traditions in particular have been investigated in recent decades (e.g., Dahl et al., 2015; Winter et al., 2020). The fact that Buddhism was thus given the image of a “scientific” religion was already expressed in the speech by Tenzin Gyatso, the 14th Dalai Lama and religious leader of a certain Tibetan Buddhist school, at the 2005 annual conference of the Society for Neuroscience in Washington, DC. In it, the Dalai Lama in turn thanked the physicists Carl Friedrich von Weizsäcker and David Bohm as well as the (neuro-) biologists Robert Livingstone and Francisco Varela for their teaching; he also referred to the Mind and Life Institute dialogs between scientists and Buddhists that have been held regularly in Dharamsala, India, since 1987.^4^ Recently, however, the ideological neutrality of the researchers involved in these initiatives and the image of Buddhism as a “scientific” religion have been called into question (Thompson, 2020). In fact, there has already been an (unsuccessful) petition against the Dalai Lama’s lecture in 2005 and some neuroscientists withdrew their participation in the conference because of the lecture (Cyranoski, 2005).

This perspective addresses the question of the compatibility of Western and yoga psychology. Since the spread of Indian yoga in Western countries, it has often been presented as a science or science-based practice (e.g., Alter, 2004; Behanan, 1937; Taimni, 1961). Of course, the effects of yoga exercises on health and fitness can be scientifically investigated, as has been done many times in recent decades, sometimes with positive results (e.g., Broad, 2012). However, can yoga *itself* provide valid insights into human consciousness and behavior in a scientific sense, as psychology and neuroscience also strive to do? In order to approach an answer to this question, we will first have to narrow down the meaning of “yoga” in the next section and then understand its epistemology. In the following section, we will deal with the conceptualization and localization of mental phenomena more generally.

## Yoga and its epistemology

“Yoga” is a word from Sanskrit, the ancient scholarly language of India. One of the (many) possible translations is “connecting link”—and this meaning can also be found in related terms in modern Indo-European languages such as the English “yoke,” Dutch “juk,” or German “Joch.” However, a glance at the Sanskrit dictionary does not give us an informative answer about yoga for our present purposes. For that, we need to understand what yoga is or was. The (rough) distinction between three historical phases has proven useful: firstly, ancient yoga as an ascetic practice to overcome the physical world and liberate the human being; secondly, medieval hatha yoga as a combination of spiritual-religious thoughts and physical exercises for a healthier life; and thirdly, the strongly body- and fitness-centered yoga of our time (see Baier et al., 2018; Singleton, 2010).

The term “yoga” has been used in Indian tradition for thousands of years. However, the nowadays most popular source are 195 aphorisms known as *The Yoga Sutras of Patanjali* whose age are estimated to date back to the first centuries of our era (e.g., Bryant, 2009; White, 2014). According to this, the yoga system is a further development of another Indian philosophical school, the Sankhya (cf. Raju, 1985), and should also be seen in the context of Buddhism, then more influential in India (Gokhale, 2020). Similar to Buddhist texts, the *Yoga Sutras* describe an eight-limbed path to spiritual liberation. In addition to ethical rules such as non-violence, honesty, cleanliness, abstinence and discipline, they also contain rudimentary instructions on postures, the well-known “asanas,” breathing exercises (pranayama) and four stages of meditation: withdrawal from sensory perceptions (pratyahara), concentration (dharana), meditation (dhyana) and finally absorption (samadhi).^5^ These rules of life, physical exercises—especially when seated—and breathing techniques are intended in particular to harden the body, calm the mind and thus support meditation. The *Yoga Sutras* can therefore best be understood as a treatise on meditation.

The text, which is (probably) more than 1,600 years old, is divided into four chapters. The first explains important basics. For our purposes, the epistemology of yoga, the sources of valid knowledge, is important here. According to the translation by Bryant (2009), the *Yoga Sutras* state: “I.7 Right knowledge consists of sense perception, logic, and verbal testimony.” The first two should be unproblematic from a scientific point of view. After all, the “empirical” in “empirical science” stands for sensory experience (from the Greek “empeiros,” to experience), which today is supported by a variety of measuring instruments. Phenomena in the world are ordered, explained or predicted through the generalization of observations using logical-mathematical procedures (cf. Chalmers, 2013). However, the third source named in the *Yoga Sutras*, verbal testimony, is not that unproblematic. This explicitly includes spiritual-religious writings from India (cf. Bryant, 2009; Gokhale, 2020). In the history of Western philosophy and science, this type of argumentation has been criticized as “argument from authority” (e.g., Rosenberg, 1996). Today, most researchers would probably find it inadmissible to base a *scientific* statement on quotations from the Bible. According to the *Yoga Sutras* however, this would be a valid statement of knowledge, whereby the validity of the ancient scriptures is derived from the (alleged) special meditative abilities of their authors, similar to the (alleged) divine inspiration of the prophets in the major world religions. In fact, the *Yoga Sutras* themselves explain that special insights arise in a state of meditative absorption (samadhi): “I.48 In that state, there is truth-bearing wisdom.” I.49 Samadhi “has a different focus from that of inference and sacred scripture, because it has the particularity of things as its object” (Bryant, 2009). According to this view, meditators then have an extraordinary perception of reality. This may be difficult to reconcile with the requirements of replicability and objectification of scientific knowledge.

Apart from the epistemology of yoga, the metaphysics adopted from Sankhya philosophy is likely to pose a major problem for compatibility with modern science. It should be borne in mind that these views can be thousands of years old and that people tried to form a picture of the world through philosophical reflection or the aforementioned meditative contemplation in the past. In the Western tradition, this would correspond to the natural philosophers, whose teachings were replaced by theories of physics, chemistry and biology, for example, in the course of the scientific revolution (cf. Schlick, 1949). According to Sankhya philosophy, all matter (prakriti) is determined by three qualities (gunas), in simple terms: harmony, motion and inertia (sattva, rajas and tamas). Interestingly, this system also includes the mental faculties, intellect (buddhi), ego (ahamkara) and perceptive mind (manas), which are all understood as *material* in nature (cf. Raju, 1985). In the yoga system derived from this, the ultimate liberation consists in the deep meditative insight that pure consciousness (purusha) is not identical with material things such as the body or the aforementioned mental faculties, but only with itself (cf. Bryant, 2009; Gokhale, 2020). In this sense, yoga has a dualistic world view, with matter and pure consciousness, whereby the ultimate goal of meditative yoga exercises is ultimately detachment from matter and absorption in pure consciousness. Perhaps the metaphysical assumptions of the Sankhya and yoga systems can be interpreted differently or metaphorically such that they can be linked to modern scientific concepts. After all, authors of these ancient scriptures did not yet know the theories and concepts of quantum mechanics or other disciplines of modern science. In a similar way, the ideas of the pre-Socratics about atoms, for example, can be transferred to the present day (cf. Chalmers, 2013). However, this endeavor is beyond the scope of this paper.

A final example of the compatibility of Western and yoga psychology can be found in the special powers (siddhis), which are described above all in the third chapter of the *Yoga Sutras* for following the path of yoga. From a scientific perspective, these include paranormal or parapsychological abilities such as invisibility, simultaneous presence in several places, clairvoyance or mind reading (see Bryant, 2009; Gokhale, 2020). Psychology in particular has distanced itself from statements about parapsychological phenomena in the course of its academic institutionalization (Hines, 2003). Therefore, the special abilities claimed for the application of the yoga system contradict today’s scientific psychology. This could be resolved by considering the powers mentioned not as real, but only as existing in the imagination, for example in states of deep meditation or when consuming mind-altering substances. The latter is widely documented in the Indian tradition and is also found at the beginning of the fourth chapter of the *Yoga Sutras*: “IV.1 The mystic powers arise due to birth, herbs, *mantras*, the performance of austerity, and *samadhi*” (Bryant, 2009). For example, it could be that the use of the substances referred to here only as “herbs” produced out-of-body experiences, which were then interpreted as real events. If such experiences are only seen as inner ideas or dreams, the corresponding statements in the yoga system would no longer contradict Western psychology or science. By contrast, they could still be investigated scientifically for their phenomenal content or therapeutic potential (e.g., Ko et al., 2022; Rechtschaffen, 1967; Scarpelli et al., 2022). This view, however, would probably imply that traditional yoga loses its authenticity: Why should the statements about meditation and liberation be taken seriously if the claims about special powers are not to be understood literally? This applies in particular to the goal of the yoga system presented here, liberation in identity with pure consciousness (purusha). Since this is by definition in contrast to the material world (prakriti), it is questionable whether it can also be scientifically researched or whether it can only be inferred from inner experience. However, if this ultimate state is only seen as an experience without metaphysical meaning, yoga loses its goal.

We have seen in this section that traditional yoga in the sense of the *Yoga Sutras* is difficult to reconcile with Western psychology or science, both at the level of epistemology and metaphysics and at the level of the abilities promised by following the path of yoga. This problem could perhaps be solved by reinterpreting old sources or concepts. However, this would make the system less authentic and meaningful. In the next section, we will briefly look at the formation and localization of concepts in Western and yoga psychology.

## Concept formation and localization

**Figure 1:**
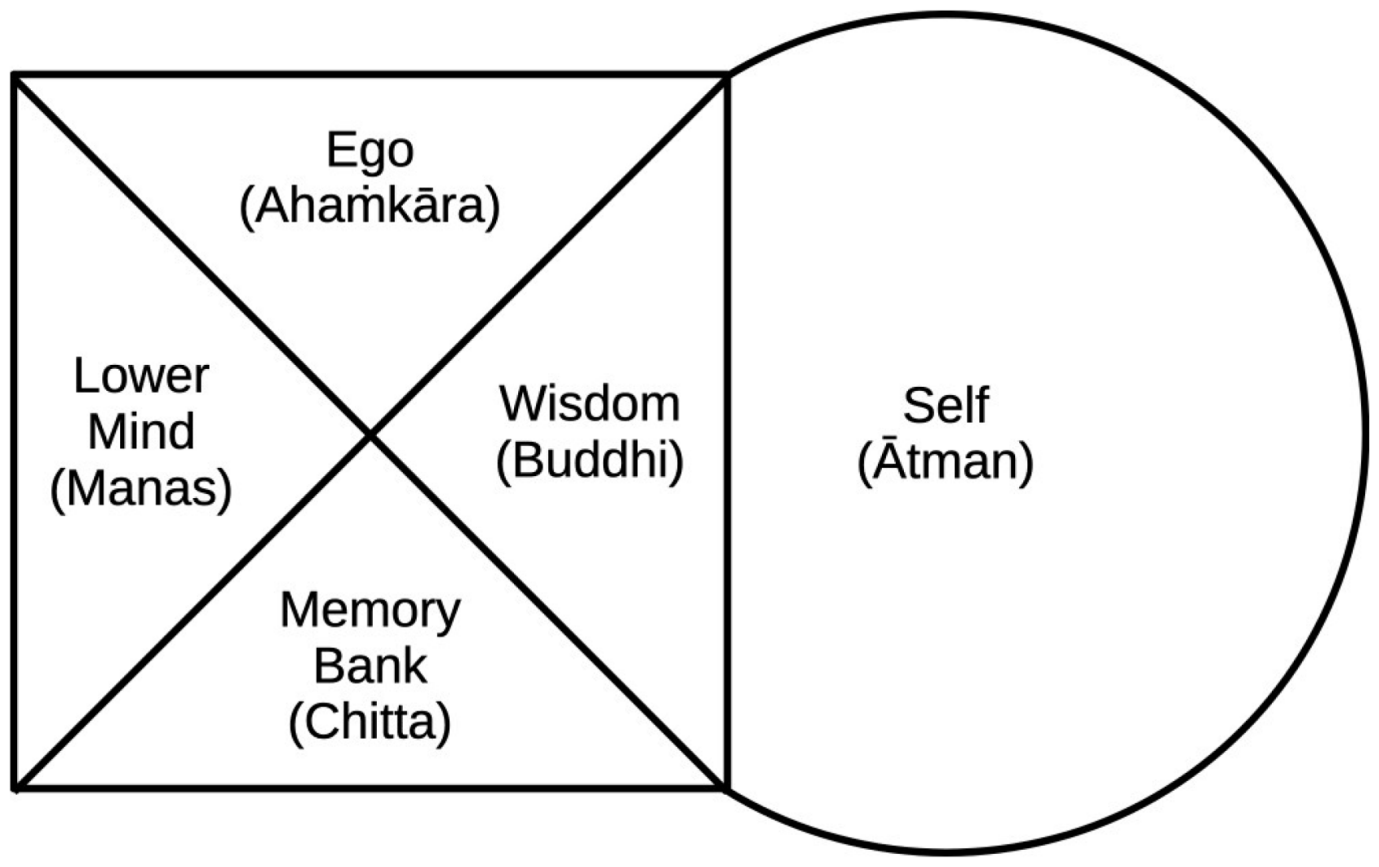
Distinction of different psychological faculties in the Indian Advaita Vedanta system: The “lower mind” is related to sense perception, the “self” a transcendental entity reminiscent of the soul in Western culture (Rama et al., 1976).

We saw in the previous section that different mental faculties were distinguished both in Indian philosophy and psychology. The *Yoga Sutras* themselves consist of 195 short verses or aphorisms complemented by explanatory commentaries, of which numerous variants have appeared over the centuries and to this day (e.g., Bryant, 2009; Gokhale, 2020; White, 2014). In addition to the Sankhya and yoga systems, a third Indian philosophical tradition in particular, Advaita Vedanta, has been related to Western psychological issues. As early as the 1970s, for example, there were collaborations between Western doctors and clinical psychologists with Indian spiritual teachers, which were also applied clinically (e.g., Rama et al., 1976). These also used a traditional Indian model of the mind which distinguishes memory (chitta) and the higher self (atman) (Figure 1).

**Figure 2:**
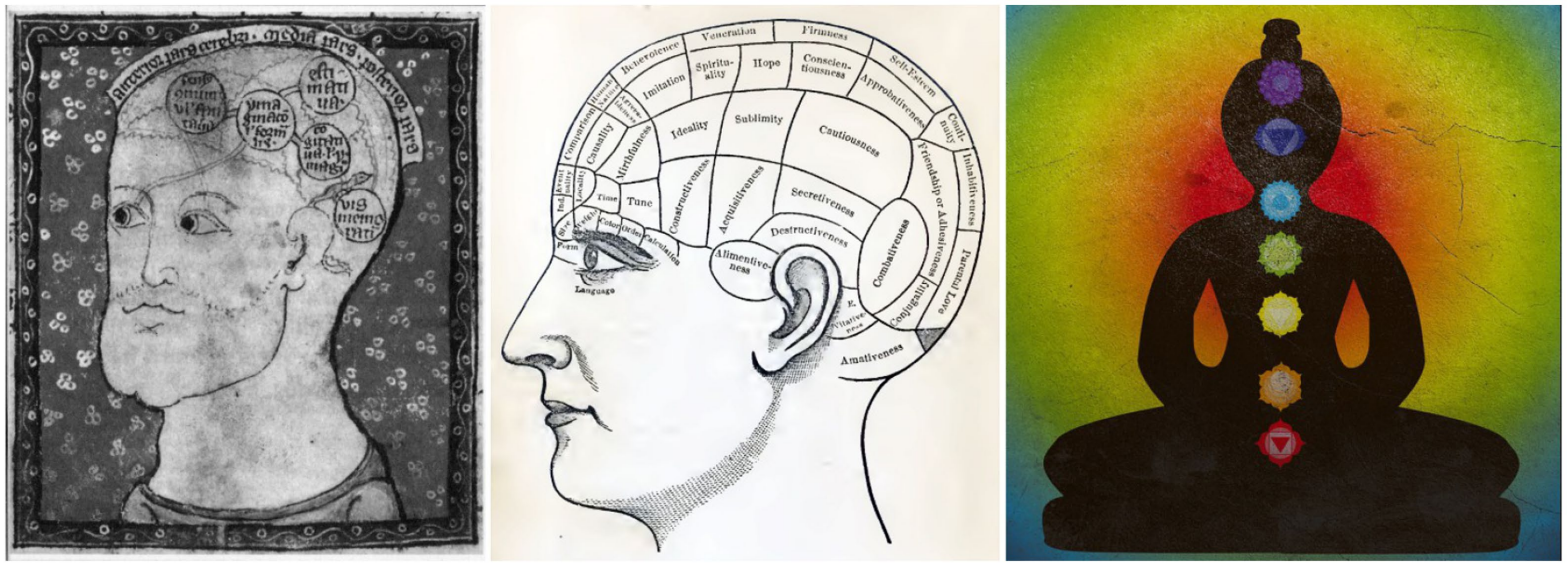
Three localizational models of the mind–body: The Arab philosopher Qusta ibn Luqa (864–923) already localized cognitive functions in the brain, here in five presumed ventricles (left); the phrenologists assumed various mental organs in the brain and on the skull (middle); the folkloristic seven-chakra model of yoga which places, for example, willpower above the navel, love in the heart area and insight between the eyes (right). Sources/License: 13th century, Cambridge University Library, van der Eijk (2008); Fowler and Fowler (1859); Alexandra Koch/Pixabay.

A more body-centered model that is popular in yoga today is that of the chakras (Sanskrit for “wheels”), which locates various “energy centers” with psychological and physical qualities in the body. In particular, the model of seven chakras located along the spine, to which the rainbow colors are assigned, has become common folklore (Figure 2). There have also been attempts to define these microbiologically (e.g., Maxwell, 2009). However, it is less well known that this seven-chakra model is only one of many from Indian philosophy and was popularized by the British theosophist Charles W. Leadbeater (1854–1934), among others (Leadbeater, 1927; cf. Eliade, 1969). In addition, there are various systems with different numbers of chakras, which are described differently; this illustrates that meditative insight can lead to different insights. For example, there is just *one* chakra, located in the navel area, mentioned in the *Yoga Sutras*: “III.29 [By meditating] on the navel plexus of the body comes knowledge of the arrangement of the body” (Bryant, 2009). Even if some scientists regard the chakras simply as “pseudoscience,” many millions of people around the world probably do exercises with them. Instead of reifying them and localizing them scientifically (e.g., Maxwell, 2009; Sadi, 2022), they could—as in the quote from the *Yoga Sutras*—be understood in the sense of a meditation exercise as a means of sharpening mind–body awareness. Then, as already described in the previous section, the corresponding experiences would not contradict science.

In the previous section we saw that traditional scriptures and meditative insights are regarded as valid sources in the yoga system, which is in conflict with scientific principles. It has now become clear that even *within* Indian philosophical currents we can find different views on human beings and their psychology. This means that we can analyze these approaches both externally, from the point of view of science, and internally, regarding their inherent consistency. To be fair, however, it should be acknowledged that the ontology of Western psychology is not unambiguous either. For example, the approaches of evolutionary or neuropsychology have not yet led to a biological anchoring of psychological processes, especially not through the neuroimaging methods whose use increased significantly in recent decades (Anderson, 2015; Noble et al., 2024; Schleim, 2025). Since the 19th century, great expectations have been associated with the localization of mental processes in the brain (Figure 2). The lack of conceptual clarity and integration is now even seen as one of the causes of the crisis in psychology (e.g., Hutmacher and Franz, 2024). In the meantime, a pragmatic operationalism prevails in experimental psychology and cognitive neuroscience: Mental processes should be defined in such a way that they lead to reproducible results in experiments—and contribute to the explanation of consciousness and behavior in the long term. Using the example of empathy (literally: “to feel in”), I showed in an earlier paper that this mental ability has been redefined in neuroimaging research from an I-Thou relation to an I-Me relation: Instead of comprehending what *another* person experienced in a certain situation, it became a question of what *oneself* would experience in this situation. This had the advantage of being able to compare a person’s brain activation with themselves, which avoided the problem of anatomical and functional variability between different brains (Schleim, 2015). The results of such experiments was suggested to teach empathy to offenders diagnosed with psychopathy—while “psychopathy” itself is a scientifically controversial concept (e.g., Lilienfeld, 1994; Rutter, 2005). We will have to face such conceptual diversity in research and practice until the ontological problem is clearly resolved in psychology and cognitive science. According to the discussion in the previous and this section, it is not likely that the yoga system or Indian philosophy more broadly can provide this ontological basis. But, of course, psychologists and scientists from other traditions also are at liberty to operationalize mental processes and thus conduct research in their ways.

## Summary and outlook

The compatibility of Western and yoga psychology is discussed in this perspective. To attempt an answer, it is first of all essential to use a clear definition of “yoga.” In the system used here, based on the *Yoga Sutras*, fundamental problems of compatibility with science emerge: In particular, the epistemic status of older scriptures and meditative insights turn out as problematic. At second glance, this is hardly surprising. After all, we would not simply regard texts from Western Middle Ages or antiquity as true without seeing them in their historical and ideological context. The described inconsistency between Western and yoga psychology could be resolved by reinterpreting the latter or understanding some of its statements metaphorically. However, this would change the central core of yoga psychology—and other streams of Indian philosophy such as Sankhya or Advaita Vedanta—and thus undermine the authenticity of these systems. The spiritual goal of yoga, liberation, its metaphysics and the special powers described are too strongly embedded in the *Yoga Sutras* and similar texts. With the chakras in particular, however, it has been shown that such a system can be influential in society even without a scientific foundation. Methods such as “Heartfulness Meditation” are also established in research and practice today (e.g., Van’t Westeinde and Patel, 2022). The extent to which the way of speaking of yoga as a science described at the beginning is then more than just marketing in the context of modern consumer culture would have to be discussed in more detail elsewhere (e.g., Jain, 2014). This result is in line with critical voices that question the portrayal of Buddhism as a “scientific religion” (Thompson, 2020).

Regardless of these challenges, meditation, mindfulness and yoga have now become established topics in scientific research (e.g., Broad, 2012; Newcombe and O’Brien-Kop, 2021). To what extent these practices have *specific* effects on fitness, wellness and health or whether they simply work in terms of general relaxation, body awareness and gymnastics requires further investigation. Various approaches are also being pursued in psychology, for example in the phenomenological tradition, to solve the ontological problem (e.g., Gutland, 2018; Wendt, 2024). In connection with neuroscientific methods, “neurophenomenology” is also trying to further consciousness research, for example by stabilizing the contents of consciousness in scientific experiments (Schleim, 2022). Whether yoga can make a decisive contribution to this remains to be seen.

## Data Availability

The original contributions presented in the study are included in the article/supplementary material, further inquiries can be directed to the corresponding author.

## Footnotes

^1^ https://www.un.org/en/observances/yoga-day

^2^ https://www.un.org/en/observances/meditation-day

^3^https://x.com/volker_turk/status/1870363607797522479 (December 21, 2024).

^4^ https://www.dalailama.com/messages/buddhism/science-at-the-crossroads

^5^It should be noted that the meaning of such ancient terms goes much deeper than can be explained in a short perspective article.
